# Effects of Indigenous Diet Iron Content and Location on Hemoglobin Levels of Ghanaians

**DOI:** 10.3390/nu12092710

**Published:** 2020-09-04

**Authors:** April Callister, Joanna Gautney, Christina Aguilar, Julian Chan, David Aguilar

**Affiliations:** 1 Department of Exercise and Nutrition Sciences, Weber State University, 1435 Village Dr., Ogden, UT 84408, USA; aprilcallister@mail.weber.edu; 2 Department of Sociology and Anthropology, Weber State University, 1299 Edvalson St., Ogden, UT 84408, USA; joannagautney@weber.edu; 3 Health, Physical Education, and Recreation Department, Weber State University, 1435 Village Dr., Ogden, UT 84408, USA; christinaaguilar@weber.edu; 4 Department of Mathematics, Weber State University, 1415 Edvalson St., Ogden, UT 84408, USA; julianchan@weber.edu

**Keywords:** Ghana, hemoglobin, anemia, iron consumption, food groups

## Abstract

Multiple studies have demonstrated strong links between diet and anemia, but few have explored the impact of food groups on hemoglobin (Hb). We analyzed the correlation between Ghanaian diet and Hb levels to explore reduction of anemia prevalence through dietary interventions. Demographics, food frequency questionnaires (FFQ), and blood samples were obtained from 140 volunteers (ages 18–65) in five locations across Ghana. Hb was measured; estimated iron consumption was calculated. FFQ items were grouped by food type, and a regression analysis was performed to determine the most important dietary predictors of Hb. Moreover, 47% of total participants were anemic; 64% of females and 28% of males. Hb levels were highest in Mole (13.9 g/dL, SD = ±1.9), independent of sex distribution. The regression model revealed a 62.7% adjusted correlation between food groups and Hb levels. Animal foods (β = 0.016, *t* = 5.08, *p* < 0.01) and plant protein (β = 0.013, *t* = 2.86, *p* < 0.01) were the most influential groups to Hb levels. It is of vital importance to emphasize the benefits of consuming animal foods and plant proteins within the Ghanaian population. The ease of access to plant proteins makes it likely that this food group will be most influential and have the greatest impact in reduction of anemia in the Ghanaian population.

## 1. Introduction

Iron deficiency is the most prevalent micronutrient deficiency worldwide, with an estimated two billion people affected: approximately 30% of the world population [1,2]. This deficiency may be caused by a variety of factors, including insufficient iron intake; periods of increased requirements; decreased absorption; or blood loss. [3]. These factors place certain populations at higher risk for iron deficiency, including women of reproductive age due to blood loss via menstruation and increased blood volume during pregnancy, as well as children, due to periods of rapid growth. [1,4,5,6,7].

Iron deficiency is the most common cause of anemia. Incidence of anemia is generally higher in third world countries, where it has been estimated that more than half of pregnant females and young children in many African countries are anemic, with estimates as high as 67.6% and 57.1% prevalence, respectively [1,2,5]. In Ghana, it is estimated that over 40% of the population is affected by iron deficiency anemia [1].

The complications of anemia are vital to address, as the effects can be felt on both an individual and a population level. Iron deficiency anemia (IDA) combines a depletion in total body iron stores and reduced iron availability to erythrocytes and other tissues [8]. Individuals may experience iron deficiency without reaching Hb levels indicative of anemia [4]. The short-term effects can include depressed immune and endocrine function; increased likelihood of infection or illness; decreased capacity for work; diminishing personal and economic achievements, and lowered oxygen transport capability. The long-term effects of anemia include pregnancy and birth complications such as low birthweight; premature delivery; perinatal complications; hemorrhage, and increased rate of maternal death. Iron deficiency during pregnancy can result in children born with a greater risk of low-iron stores and impaired physical and cognitive developments [1,7]. Bailey et al. proposed that the cyclic nature of micronutrient deficiencies, including iron, have intergenerational consequences. Children born to mothers with deficiencies have a higher risk of low stores or deficiencies at birth, and a reduced capacity to increase stores, due to the low availability of the micronutrient [1].

It is of vital importance to find sustainable, long-term solutions to reducing prevalence of anemia worldwide. The solutions of highest impact will be those tailored to specific populations, where the needs of the individuals are considered. As iron is a fundamental part of the human diet, one of the most sustainable methods to addressing iron deficiency and IDA is to address the dietary needs of individual populations, and look for long-term solutions within the current diet.

The sources of iron available in the diet include heme, sourced from animal foods, and nonheme, derived from plant, animal, and fortified foods [9]. It is estimated that heme iron is absorbed at a rate three times higher than nonheme iron, making it a far more bioavailable source [10]. Heme iron absorption is not affected by dietary enhancers, inhibitors, or body iron stores. Factors that can enhance absorption of nonheme iron include a peptide present in meat, fish, and poultry, known as MFP Factor, and ascorbic acid (vitamin C), which works through iron chelation and reduction. Inhibitors of nonheme iron include phytates, found in lentils, soybean, and unrefined rice and grains, polyphenols, found in tea and red wine, and calcium [2]. The Ghanaian diet varies nationwide, but some macro- and micronutrient patterns have been noted in the literature with respect to rural versus urban populations. Largely, the Ghanaian diet relies on fruits, cereals, and starchy roots, with starchy roots comprising nearly three quarters of dietary energy in most populations [11]. Beyond this generalization, studies have shown carbohydrate consumption to be higher in rural areas than in urban areas, while protein and fat consumption tends to be higher in urban than rural communities [12].

Galbete et al. demonstrated that a large percentage of Ghanaians living in rural areas consumed a diet rich in nuts, seeds, roots, tubers and plantains, fermented maize products, legumes, and palm oil. In urban areas of Ghana, diets were more frequently high in dairy products, red and processed meats, eggs, legumes, rice and pasta, and meaty mixed dishes [12]. Among mothers in rural Ghana, it was reported that only 51% of individuals consume meat (non-poultry, unspecified type) daily or weekly. Fish was consumed on a daily or weekly basis by 97.8% of the study population, while poultry was consumed occasionally (less than once per month) by 95% of individuals. Non-heme sources of iron, such as maize, groundnut and cowpea were consumed on a daily or weekly basis by 98.6%, 83.5%, and 74.8% of the population, respectively [13]. These studies aid our understanding of the dietary components and eating habits that may influence and individual’s risk for iron deficiency and IDA, but specific recommendations for the Ghanaian population to increase iron in the diet are currently lacking. Also lacking are data on the effects of specific food groups on iron levels in the body, which will aid in developing more specific recommendations for the population.

This study aims to assess incidence of anemia and the role that local diet patterns play in that prevalence. We hypothesized that individuals who regularly consumed a variety of iron-rich foods would have higher Hb levels than those who consumed a less iron-rich diet. In addition to this, we sought to characterize the influence of specific food groups on the Hb levels of this population.

## 2. Materials and Methods

### 2.1. Location

Our study (IRB# 16-ED-042) was designed as a cross sectional sample, including five areas in Ghana: Jamestown, Kumasi, Larabanga, Mole, and Accra. Data was collected from 140 volunteers ages 18–65, comprised of 76 females and 64 males. One-day clinics were set up at each location; researchers spoke with all willing attendees and asked each to participate in the study procedures. After explaining study procedures, written consent was obtained by the PI, Co-PI, and/or trained assistants; subjects who did not know how to sign their full name were asked to sign an X on the line for their signature, and the data collector provided a witness signature.

### 2.2. Data Collection and Analysis Methods

Data collected included demographic information: age, education, marital status, main occupation, income; food intake and disease information; number of meals per day, meals at home, meals outside home, knowledge of sickle cell disease, most recent malarial infection, food frequency questionnaires (FFQ) and blood samples. FFQ were designed based on previous studies done in Ghana, by Nti et al., Agyemang et al., Abubakari et al. as well as the 2014 Ghana Demographic and Health Survey [12,13,14,15]. A three-day diet record was collected from 20 study participants, with the purpose of providing more detailed information to standardize the diet record using calorie consumption [16]. Blood samples were obtained using a single fingerstick method with Alere Hemopoint H2 Meter 55,118 (enzyme spectrophotometric method) to assess Hb and hematocrit levels as biomarkers of anemia status. The data collection and statistical analysis in this paper correspond to a power of greater than 95% for the indicated statistical tests and corresponding sample sizes in the analysis. This indicates that the data collection method and analysis are appropriate for the scope of the study.

### 2.3. Statistical Analyses

All statistical analyses were performed with the R-studio program for statistical computing and graphics © 2009–2018 RStudio (V.1.1.463), Inc. (250 Northern Ave, Boston, MA 02210, USA). The package: “readxl” (V. 1.2.0), and “pwr” (V. 1.2.2) were used in the statistical analysis. Anemia prevalence was determined with 95% confidence intervals (CI), and associations between dietary patterns and iron biomarkers were determined by multivariate analysis. Odds ratio (OR) tests were performed to estimate the magnitude of the association between dietary patterns and iron levels.

## 3. Results

### 3.1. Anemia Prevalence and Hemoglobin Levels by Geographic Location

The prevalence of anemia observed in this population according to the World Health Organization (WHO) guidelines was 47%, with 64% prevalence in females and 28% in males [17]. Hb levels varied between the five locations. The area with the highest average Hb was Mole (13.9 g/dL, SD = ±1.9), followed by Accra (13.2 g/dL ± 1.9), Kumasi (12.7 g/dL ± 2.3), Jamestown (12.1 g/dL ± 1.9), and Larabanga (11.9 g/dL ± 1.9) (see Table 1). A two-way ANOVA test was performed to analyze if differences between towns were due to variable sex distributions; analysis revealed that location was still significant with a *p*-value of 0.01, and that sex was significant with *p*-value of 3 × 10^−14^. A post-hoc test showed that Hb levels in Mole were significantly different than levels in Larabanga (*p*-value = 0.03), with a difference of 1.3 g/dL and 95% confidence interval (0.05, 2.54), as well as Jamestown (*p*-value = 0.02), with a difference of 1.15 g/dL and 95% confidence interval of (0.07, 2.22). Differences between Mole and Kumasi, as well as Mole and Accra, were not statistically significant.

### 3.2. Difference by Sex in Hemoglobin Levels

Next, we investigated the incidence of Hb level variation between sexes. The total study population of males and females were found to have different overall Hb levels (*p*-value ≈ 7 × 10^−15^), with an estimated difference of 2.63 g/dL, with 95% confidence interval ranges from (2.04, 3.22) (Table 2).

### 3.3. Associations between Hemoglobin Levels and Dietary Iron Consumption

**Figure 1:**
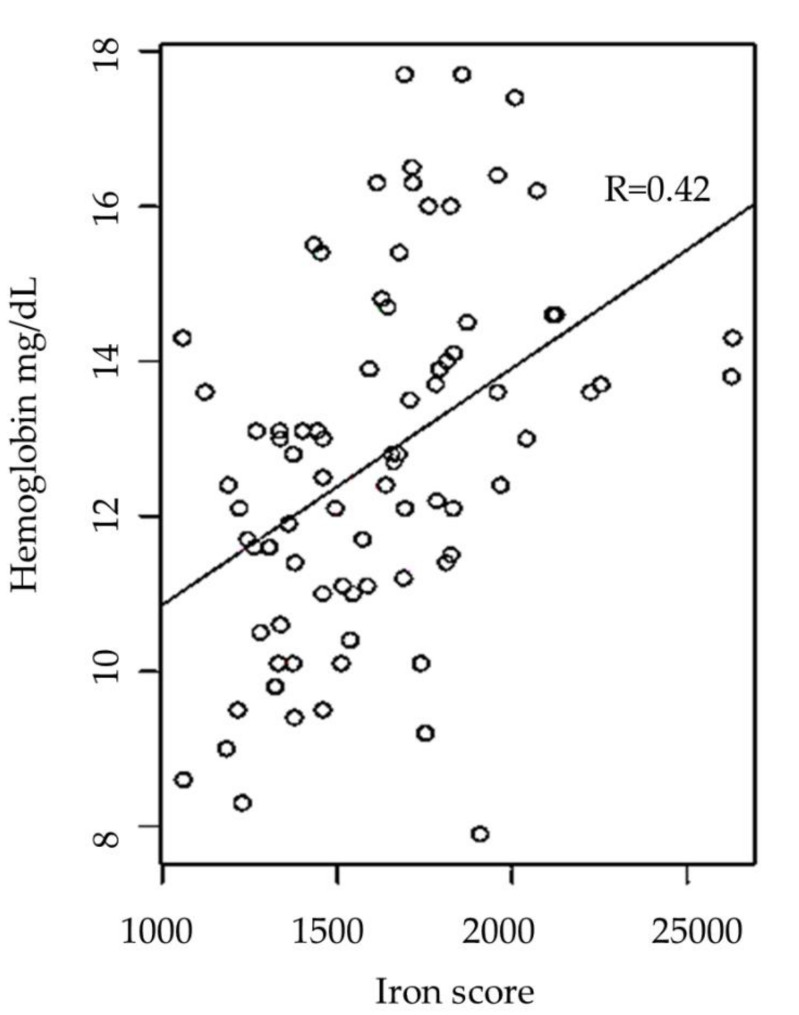
Correlation between hemoglobin and iron score. A moderate positive association was observed between hemoglobin circulating levels and participants iron consumption score *n* = 140, *R* = 0.42 and *p* = 8 × 10^−5^.

Our third data analysis investigated the claim that iron consumption affects Hb levels. An individual iron score for each person was calculated based on the average calories distributed across the foods indicated on the FFQ (see method below), and the iron content category of the food; this was done to standardize the amount of calories consumed, and to control for variance in responses. A regression on Hb levels vs. iron score was performed, and a correlation coefficient of 42.9% was observed (*p*-value = 8 × 10^−5^) (see Figure 1)

The process to configure iron score was as follows:

A. Time variables of consumption of each food on the FFQ included daily, weekly, monthly, occasionally, and never. Each time variable was given a numerical value to stratify the data proportionally according to frequency of intake. The numerical values were as follows: daily = 1; weekly = 3/7; monthly = 1/10; anything consumed on less than a monthly basis was given a value of 0.
B. The data were then split by sex, with males given a total caloric allotment of 1300 calories per day, and females 1000 calories per day. The estimates of 1000 and 1300 calories a day for females and males, respectively, was based upon 3-day diet recalls that were collected from 20 individuals (females = 1014 ± 56 kcal males = 1311 ± 78 kcal).
C. The individual’s weighted intake of food was calculated by summing the values of each food’s time variable value codes (part A). This gave each person a single number that represented the “weight” of food in the diet. Larger numbers, or higher score, implied higher frequency of intake of a more diverse diet.
D. The weighted average calorie intake per food, based on allotted calories for the individual sex, was then calculated by dividing total calories by weighted individual intake (B/C).
E. Each food’s time variable code (part A) was then multiplied by the average calorie per food (part D) (A × D). This gave a proportional estimate of how much influence a single food had in an individual’s total caloric intake.
F. Each food’s individual proportional value (part E) was then multiplied by the iron score (see Table 3). Values were summed for every food in the diet to give a total iron score (single numerical value). The iron score of each food was determined by ranking foods from 0–4, (where 4 = very high source > 20% of RDA/serving; 3 = high source 10–20% RDA/serving; 2 = good source 5–10% RDA/day; 1 = low source < 5% RDA/serving; 0 = not a source); the score was calculated based on amount of iron present per serving size.

### 3.4. Regression Analysis of Hemoglobin Diatery Determinants

Next, we grouped the foods according to food group and calculated the total calories an individual consumed from each food group. A regression model was performed for Hb based on the number of calories consumed from each food group. The result was a correlation of 67% and adjusted correlation of 62.7%. The regression model demonstrated that animal foods contribute the most per calorie towards Hb levels with 0.0168 Hb level for every calorie of animal food consumed (*p*-value = 2.9 × 10^−6^). The *p*-value of animal foods was smaller than any other coefficient for food groups, though all foods contributed to some level (see Table 4). A regression equation was generated (see below) based on the regression model.

Regression Equation

Hb = 0.42 + 0.016 × animal + 0.005 × starch + 0.012 × plant protein + 0.011 × vegetables + 0.010 × fruits + 0.010 × oil + 0.007 × drink

### 3.5. Differences between Low and High Iron Consumers

To investigate the difference in Hb levels between high and low-iron consumers, individuals were classified in the following way. Daily and weekly consumption of foods were taken into consideration. A multiplier of 7 was applied if a food was consumed daily, and a multiplier of 1 if the food was consumed weekly. Any other time, the variable was given a score of 0. The same unit values from 0 to 4 based on level of iron content were used where 4 = very high source > 20% of RDA/serving; 3 = high source 10–20% RDA/serving; 2 = good source 5–10% RDA/day; 1 = low source < 5% RDA/serving; 0 = not a source. A total score was calculated based on frequency of intake of iron containing foods. This was based on the recommended daily allowance (RDA) for iron, of 18 mg for females and 8 mg for males. Finally, to create the dichotomy of high and low iron consumers, a threshold of at least 80% RDA was established as the minimum amount of iron in the diet required to be considered a high-iron consumer. This threshold was chosen to allow for a round number of iron-containing foods consumed per day (2 in males and 4 in females). For males, a score of 56 indicates consumption of a very high iron content food (worth 4 units), two times per day, seven days per week (4 × 2 × 7 = 56). For females, a score of 112 indicates consumption of a very high iron content food (worth 4 units), four times per day, seven days per week (4 × 4 × 7 = 112). It is also possible to combine various levels of iron content in foods with various frequencies of intake, as long as the threshold of 56 units and 112 units is achieved. 

Analysis demonstrated that participants classified as high iron consumers showed a trend of higher Hb levels (12.75 mg/dL ± 2.8 vs. 10.73 ± 3.5 mg/dL) when compared to their lower iron intake counterparts (*p* = 0.07).

## 4. Discussion

Our study explored the relationship between local diet and iron levels in the Ghanaian population, with an aim to develop population-specific guidelines to address IDA in the community. The development of guidelines that adhere closely to the community’s current diet have a higher chance of making long-term improvements in the diet, as less drastic changes are needed and can be more seamlessly integrated into current dietary habits. The results of our study showed the anemia prevalence in the 140 participants was 47%. As our study population did not exclude based on age or sex, our recommendations can be more inclusive to the entire population. Using the work of studies by Nti, Galbete, Tata, and others, alongside our own data, will help us to establish concrete dietary recommendations for the study population based on the local dietary patterns. Our study is one of the first to establish concrete data analyzing the influence of current local foods on Hb levels, a vital component of creating dietary recommendations that are sustainable, accessible, and customizable to the individual [7,12,13].

### 4.1. Location and Hemoglobin

The variance of Hb levels based on location is suspected to be influenced by geographic location and access to game meat. A similar study by Tata found that proximity to forested areas in Southwest Cameroon were associated with higher Hb levels, presumably due to greater access to various fruits, vegetables, and animal foods [7]. Though proximity to forested areas was not considered in our study design, Tata’s findings are suggestive that similar factors could be at play in our results. Analogous patterns between dietary diversity, micronutrient deficiencies such as IDA, and proximity to forested areas have been observed world-wide, especially in tropical regions [7,18,19,20].

This idea was reinforced when sex was factored in and results remained significant, ruling out the possibility that certain locations had a disproportionately large number of male or female participants that skewed results. As animal foods were the food group of highest influence on Hb levels at 0.0168 Hb levels per calorie of animal food, areas with greater access to a wide variety of animal foods are likely to have higher consumption on a regular basis, and therefore higher intake of heme iron, which would heavily influence Hb levels. This principle is likely influential in Ghana. The study location of Mole is in close proximity to Mole National Park, Ghana’s largest wildlife refuge. While trophy poaching is a great threat to most of Africa’s other wildlife refuges, poaching at Mole National Park is indiscriminate. Authorities report the vast majority of poaching at Mole National Park is either for the bush meat trade or is local subsistence poaching [21,22]. It is unsurprising that the Mole group should have the highest Hb levels among those studied, given Ghana’s cultural emphasis on bush meat consumption paired with the proximity of the national park.

The purpose of determining iron score within the realms of total caloric intake was to control for skewed results from the FFQ. Due to the nature of the FFQ, the statistical analysis of an individual with greater dietary diversity would have falsely implied greater calorie and iron intake than an individual with lower food variety consumption [23]. Because it is difficult to calculate caloric intake from an FFQ, the total daily caloric intake limit of 1000 calories a day for females and 1300 calories a day for males was not created with the goal of individual accuracy, but rather to predict the proportion of iron from a given food in the diet for the individual [16]. To control for the fact that males generally consume greater amounts of food than females and therefore are likely to have more iron in their diets, the caloric standard of 1000 calories per day for females and 1300 calories per day for males was assigned to each sex. The use of striating the frequency of intake proportionally was also used to overcome variances in intake. The use of units of daily = 1, weekly = 3/7, monthly = 1/10, and occasionally = 0 were based upon the assumption that a food consumed 4–7 × per week could be considered daily, so a ratio of 7/7 (1) was used; a food consumed 1–3 times per week could be considered weekly, so a ratio of 3/7 was used; a food consumed 1–3 times a month could be considered monthly, so a ratio of 3/30 (1/10) was used. It was assumed that foods eaten less frequently than once per month were not influential enough in the diet to be considered.

An iron factor of 0–4 units was used rather than the actual amount of iron in the food to stratify the data into categories of iron consumption; because it is impossible to estimate individual portion sizes based on the FFQ, it would be less accurate to use the actual amount of iron present in a single serving of the food. Classifying the foods on a scale of 0–4, or absent to high in iron, was a more accurate way to process the data when considering the RDA for iron.

The regression analysis showed consumption of animal foods to be the most important dietary factor influencing hemoglobin levels (β = 0.016, *t* = 5.08, *p* < 0.01). This has been observed in multiple previous studies [7,24,25]. However, our study shows that plant protein sources commonly consumed in this population, such as cowpeas, soybeans, groundnuts, bambara, agushie and neri also play an important role in the modulation of hemoglobin levels (β = 0.013, *t* = 2.86, *p* < 0.01). This is an important observation because plant sources may be more accessible and affordable to a greater majority of the population. Examples of this can be found in common local dishes such as waakye and bambara soup, which contain pulses that are high in iron. Focusing on the increased consumption of these types of food items may be a more realistic and effective strategy for increasing the hemoglobin levels of this population, since they are already an intrinsic part of the diet. Finally, it was observed that fruit and vegetable consumption influenced this population’s Hb levels to some degree. Many fruits and vegetables consumed in Ghana, such as peppers, citrus, pineapples, etc., are high sources of Vitamin C. Ascorbic acid is a potent enhancer of non-heme iron absorption due to its function as a reducing agent, which increases solubility and absorption of ferrous iron in the duodenum [26]. It is very likely that the impact observed by these food groups in Hb levels is associated with this effect.

### 4.2. Differences between High Iron and low Iron consumers

Our study allowed us to identify practical guidelines for the consumption of foods that are linked to higher Hb levels and the prevention of IDA. We observed that the daily consumption of the equivalent of two high iron food sources (or a score of 56 units) in males and four high iron food sources (or a score of 112 units) in females, was associated with higher Hb levels. This model aids in the establishment of practical, flexible dietary recommendations for the prevention of IDA based on foods that are regularly consumed in these communities. Our study can be used to establish the framework for an iron intake exchange system. This system would be based on a goal of 56 units in males and 112 for females, from a variety of food equivalents in the Ghanaian diet. Individuals who may not be able to consume very high iron sources may be able to increase their consumption of moderate iron sources to prevent low Hb levels.

## 5. Conclusions

As noted above, it is of vital importance to reduce the prevalence of anemia worldwide and in Ghana specifically, and the most impactful solutions will be those tailored to individual populations [27]. This study highlights the truth of this concept. Effective solutions to the global epidemic of anemia should be tailored not only from region to region or country to country, but to individual populations within those countries. Despite the nation’s relatively small geographic size, our data show a marked difference in anemia prevalence between some of the groups studied within Ghana. This has shown to be true for other nutrition-related health issues as well. The low levels of hemoglobin in this population may be indicative of low iron intake, but could also be indicative of poor diet quality overall. In addition to anemia including IDA, Ghanaians are at risk for a number of nutritional deficiencies and diseases, including calcium and B-vitamin deficiencies as well as type 2 diabetes; yet studies have shown that there is no “one-size-fits-all” approach to alleviating these problems [13,28,29]. Nutrition education initiatives aimed at increasing consumption of locally available foods rich in iron and vitamin C (and other micronutrients that are lacking in diets) should be undertaken to empower the local population and decrease the prevalence of iron deficiency anemia in Ghana.

## Figures and Tables

**Table 1:** Average hemoglobin of the five study locations, with standard deviation.

Locations	Jamestown	Kumasi	Larabanga	Mole	Accra
Mean Female Hemoglobin (mg/dL)	11.4 ± 1.7	10.9 ± 2.0	11.2 ± 1.7	10.4 ± 1.1	12.1 ± 0.6
Mean Male Hemoglobin (mg/dL)	13.6 ± 1.6	14.0 ± 1.4	13.0 ± 1.8	14.5 ± 1.8	14.7 ± 2.0
Overall Mean Hemoglobin (mg/dL)	12.1 ± 1.9	12.7 ± 2.3	11.9 ± 1.9	13.2 ± 2.5	13.3 ± 1.9

All values are means +/− Standard deviations. Data show the average Hb levels of each location, Jamestown *n* = 56, Kumasi *n* = 12, Larabanga *n* = 28, Mole *n* = 30, Accra *n* = 12.

**Table 2:** Mean and standard deviation of hemoglobin by sex.

Sex	*n*	Mean	Median	Standard Deviation
Female	76	11.3	11.4	1.6
Male	62	13.9	13.7	1.8

Average Hb based on sex, with standard deviation. Females showed lower hemoglobin levels *p* ≥ 0.05.

**Table 3:** Iron content and score value assigned to each food in the food frequency questionnaire.

Food	Iron Content	Normal Serving Size	Heme/Nonheme	Iron Score
Cassava	0.343 mg	1 cup, diced	N	1
Yam	0.81 mg	1 cup, diced	N	1
Cocoyam	0.572 mg	1 cup	N	1
Sweet Potato	0.36 mg	0.75 cup	N	1
Plantain	1.16 mg	1 cup, diced	N	2
Maize	4.5 mg	1 cup	N	1
Rice Pasta	0.53 mg	100 g	N	1
Millet	6.02 mg	1 cup	N	1
Bread Biscuits	2.4 mg	100 g	N	1
Meat	3.6 mg	3 ounces	H	4
Fish	1.1 mg	3 ounces	H	4
Poultry	0.81 mg	3 ounces	H	4
Egg	0.766 mg	1 medium (44 g)	H	1
Snail	2.98 mg	3 ounces	N	1
Cowpea	4.5 mg	1 cup	N	4
Soybean	9.2 mg	1 cup	N	4
Groundnut	6.69 mg	1 cup	N	4
Bambara	3.96 mg	1/2 cup	N	4
Agushie	0.36 mg	1/2 cup	N	1
Neri	0.72 mg	1 cup	N	1
Orange	0.18 mg	1 cup	N	1
Mango	0.26 mg	1 cup	N	1
Pineapple	0.478 mg	1 cup, chunks	N	1
Pawpaw	0.18 mg	100 g	N	1
Banana	0.39 mg	1 cup	N	1
Watermelon	0.365 mg	1 cup	N	1
Tomato	0.486 mg	1 cup	N	1
Onion	0.08 mg	0.25 cup, chopped	N	1
Leafy Vegetables	0.813 mg	1 cup	N	2
Okro	0.62 mg	1 cup	N	1
Garden Eggs	0.189 mg	1 cup	N	1
Pepper	0.36 mg	1 pepper	N	1
Refined Vegetable Oil	0 mg	1 tbsp	N	0
Palm Oil	0 mg	1 tbsp	N	0
Groundnut Oil	0 mg	1 tbsp	N	0
Coconut Oil	0 mg	1 tbsp	N	0
Margarine or Shea Butter	0 mg	1 tbsp	N	0
Palm Fruits	0 mg	100 g	N	0
Cakes and Sweets	0 mg	1 cup	N	0
Soda	0 mg	1 cup	N	0
Juice	0.2 mg	1 cup	N	1
Coffee and Tea	0 mg	1 cup	N	0
Milk	0 mg	1 cup	H	0
Alcohol	0 mg	1 cup	N	0

Iron score value of each food was based upon amount of iron/serving size. Iron content of foods were obtained from the USDA Nutrient Data Laboratory. The value as an iron source: 4 = very high source, 3 = high source, 2 = good source, 1 = low source, 0 = not a source. Score calculated based on amount of iron present per serving size.

**Table 4:** Hemoglobin regression analysis.

Variable	Regression Coefficient	Standard Error	*t*-Value	*p*-Value
Constant	0.42	1.6	0.35	0.72
Animal	0.016	0.003	5.08	2.94 × 10^−6^ **
Starch	0.005	0.002	2.28	0.02 *
Plant protein	0.012	0.004	2.86	0.005 **
Vegetables	0.011	0.002	4.42	0.02 *
Fruits	0.010	0.003	3.64	3.52 × 10^−5^ **
Oil	0.010	0.004	2.49	0.01 *
Drinks	0.007	0.003	1.87	0.061990

The regression model showed an adjusted correlation of 67.2%, with a residual standard error of 1.77, Multiple *R*-squared of 0.448 and adjusted *R*-Squared of 0.393. * Correlation is significant at the 0.05 level (two-tailed). ** Correlation is significant at the 0.01 level (two-tailed).
